# Adaptive Critic Learning-Based Robust Control of Systems with Uncertain Dynamics

**DOI:** 10.1155/2021/2952115

**Published:** 2021-11-16

**Authors:** Jun Zhao, Qingliang Zeng, Bin Guo

**Affiliations:** ^1^College of Mechanical and Electronic Engineering, Shandong University of Science and Technology, Qingdao 266590, China; ^2^College of Transportation, Shandong University of Science and Technology, Qingdao 266590, China

## Abstract

Model uncertainties are usually unavoidable in the control systems, which are caused by imperfect system modeling, disturbances, and nonsmooth dynamics. This paper presents a novel method to address the robust control problem for uncertain systems. The original robust control problem of the uncertain system is first transformed into an optimal control of nominal system via selecting the appropriate cost function. Then, we develop an adaptive critic leaning algorithm to learn online the optimal control solution, where only the critic neural network (NN) is used, and the actor NN widely used in the existing methods is removed. Finally, the feasibility analysis of the control algorithm is given in the paper. Simulation results are given to show the availability of the presented control method.

## 1. Introduction

The basis of intelligent optimization decision-using adaptive dynamic programming (ADP) method is the optimal control design. There are many mature methods for optimal regulation control design of linear systems in the field of control theory and control engineering. However, for general nonlinear systems, Hamilton–Jacobi–Bellman (HJB) equation is yielded. The analytical solution of HJB equation is not easy since it is inherently a partial differential equation. Recently, the optimal control design of systems has attracted extensive attention. Among them, the successive approximation methods [1–3] overcome this difficulty via finding the approximate solution of HJB equation, which is closely related to the ADP method. ADP is a new method based on the idea of intelligent learning, which can provide effective optimal control solution for complex dynamic systems [4, 5]. In the past two decades, ADP has been widely used in solving adaptive optimal control problems of discrete-time and continuous-time systems [6, 7]. Now, data-driven control design has become a research hotspot in the field of control theory and control engineering [8, 9]. The ADP methods can promote the research of data-based decision-making and optimal control and is conducive to the development of artificial intelligence and computational intelligence technology.

Most of the existing results of ADP methods are obtained without considering the uncertainty of the controlled plant. However, the actual control system is always affected by model uncertainty, external disturbance, or other changes. We must consider these factors in the controller design to avoid the deterioration of the performance for the closed-loop system and improve the robustness of the controlled system. For robust control design, several alternative methodologies have been suggested in the control community. The work in [10] exploited the relationship between the robust control and the optimal control of nominal system subject to a specific value function. It indicates that one can design a robust control by solving an equivalent optimal control problem alternatively. Similarly, it was shown in [11] that the robust control design may be accomplished by addressing an *H* control problem. Nevertheless, online solving the derived optimal control equations was not discussed in [10]. Instead, they adopted offline schemes to seek for the solution of the derived optimal control equations. Recently, robust control design using the adaptive critic learning method has gradually become one of the research hotspots in the field of ADP, and many methods have been proposed [12–14]. These results fully show that the ADP method is suitable for robust control design of complex nonlinear systems in uncertain environment. Since many previous ADP literatures do not focus on the robust performance of the controller, the emergence of robust adaptive critic control greatly expands the application scope of the ADP method. Generally, the controller based on robust ADP can not only stabilize the original uncertain system but also make the system optimal without dynamic uncertainty. Thus, adaptive critic learning-based robust control includes the discussion of system stability, convergence, optimality, and robustness. It plays an important role in the field of intelligent learning control of complex systems in uncertain environment.

Based on the above facts, we develop an adaptive critic learning algorithm to resolve the robust control problem of uncertain systems. To this end, we construct an equivalence between the robust control problem and the optimal control problem via selecting the appropriate cost function; then, a single critic NN is used to reformulate the cost function. To realize the optimal control solution, we design an adaptive critic leaning algorithm; since it has strong convergence, the actor NN widely used in existing ADP results is removed. Then, the feasibility analysis of the control algorithm is also given in the paper. Simulations are given to indicate the validity of the developed method.

The major contributions of this paper include

(1). To address the robust control problem, we transform the robust control problem of uncertain systems into an optimal control problem of the nominal system. It provides a new approach to address the robust control problem.
(2). Different to [13], the uncertainty in the input matrix is considered in this paper, and then, the proposed control method is used in robotic systems. This helps to apply the proposed control algorithm to the practical industrial robotic systems in the future.
(3). A novel designed adaptation algorithm driven by the NN weights' errors is used to online learn the critic NN weights. Different to [15], the convergence of the estimated NN weights to the true values can be retained.

This paper is organized as follows. In Section 2, we introduce the robust control problem and transform the robust control problem into an optimal control problem. In Section 3, a single critic NN is used to reformulate the optimal cost function, and then, an adaptive critic learning method is proposed to address the derived optimal control problem.  Section 4 gives some simulation results to illustrate the effectiveness of the proposed method. Some conclusions are stated in Section 5.

## 2. Preliminaries and Problem Formulation

A continuous-time (CT) uncertain system can be written as

(1) $$\begin{array}{c} \dot{x}\left(t\right)=f\left(x\right)+g\left(x\right)\left(u+b\left(x\right)u\right)+g\left(x\right)d\left(x\right), \end{array}$$

where *x* ∈ *ℝ*^*n*^ and *u* ∈ *ℝ*^*m*^ are the system state and the control action, respectively. *f*(*x*) ∈ *ℝ*^*n*^ with *f*(0)=0 and *g*(*x*) ∈ *ℝ*^*n*×*m*^ are the nonlinear functions. *b*(*x*) and *d*(*x*) are the uncertainties. The purpose of this paper is designing a controller to make system (1) asymptotically stable under the uncertainties *b*(*x*) and *d*(*x*). To this end, we give following assumptions.

**Assumption 1 .**

*b*(*x*) ≥ 0 is the uncertainty in the input matrix. The uncertainty *d*(*x*) is bounded, i.e., there exists a nonnegative function *d*_max_(*x*) such that ‖*d*(*x*)‖ ≤ *d*_max_(*x*).

To design a robust controller for the linear system, a linear matrix inequality (LMI) is proposed [16], while for nonlinear system (1), it is not easy. Inspired by [10, 12], an equivalence is built between the robust control problem of the uncertain system and the optimal control of the nominal system via selecting the appropriate cost function. Thus, we define the nominal system of the uncertain system (1) as

(2) $$\begin{array}{c} \dot{x}\left(t\right)=f\left(x\right)+g\left(x\right)u. \end{array}$$

For system (2), a control action *u* should be found to minimize the following cost function [17]:

(3) $$\begin{array}{c} V\left(x\right)=\int_{t}^{\infty }\left(d_{\max}^{2}\left(x\right)+x^{T}Mx+u^{T}Nu\right)\text{d}s, \end{array}$$

where *M* ∈ *ℝ*^*n*×*n*^ and *N* ∈ *ℝ*^*m*×*m*^ are the positive definite weight matrices. Hence, based on the optimal principle, we can obtain the Lyapunov function of the cost function (3) as

(4) $$\begin{array}{c} 0=V_{x}^{T}\left(f\left(x\right)+g\left(x\right)u\right)+d_{\max}^{2}\left(x\right)+x^{T}Mx+u^{T}Nu, \end{array}$$

where *V*_*x*_=∂*V*/∂*x* is the derivative of *V* with respect to *x*.

Therefore, we can get the optimal cost function as

(5) $$\begin{array}{c} V^{*}\left(x\right)=\min V\left(x\right), \end{array}$$

and its corresponding HJB equation can be given as

(6) $$\begin{array}{c} 0=\min H\left(x,u^{*},V^{*}\right). \end{array}$$

By solving (6), we have the optimal control action as

(7) $$\begin{array}{c} u^{*}=-\frac{1}{2}N^{-1}g^{T}\frac{\partial V^{*}\left(x\right)}{\partial x}. \end{array}$$

Then, we will give the lemma to explain the robust control problem of system (1) which can be transformed into an optimal control problem of system (2) via constructing cost function (3).

**Lemma 1 (see [11, 18]).**

Assume that the solution can be solved via optimal control problem of system (2) with cost function (3) and *d*_max_^2^(*x*) ≥ *d*^*T*^(*x*)*N*  *d*(*x*), and this solution can make uncertain system (1) asymptotically stable, which means that the optimal control solution is the solution of the robust control problem for system (1).

**Proof**

Because *V*^*∗*^(*x*) > 0 for *x* ≠ 0 and *V*^*∗*^(0)=0 for *x*=0 given in (5), then we can consider *V*^*∗*^(*x*) is a Lyapunov function; based on (6) and (7), we have

(8) $$\begin{array}{c} {\dot{V}}^{*}\left(x\right)=V_{x}^{*T}\dot{x}\\ =V_{x}^{*T}\left(f\left(x\right)+g\left(x\right)\left(u^{*}+b\left(x\right)u^{*}\right)+g\left(x\right)d\left(x\right)\right)\\ =-d_{\max}^{2}\left(x\right)-x^{T}Mx-u^{*T}Nu^{*}+V_{x}^{*T}g\left(x\right)b\left(x\right)u^{*}+V_{x}^{*T}g\left(x\right)d\left(x\right)\\ =-d_{\max}^{2}\left(x\right)-x^{T}Mx-u^{*T}Nu^{*}-2u^{*}{\;}^{T}N\;d\left(x\right)-2u^{*}{\;}^{T}Nb\left(x\right)u^{*}\\ \leq -d_{\max}^{2}\left(x\right)-x^{T}Mx-u^{*T}Nu^{*}-2u^{*}{\;}^{T}N\;d\left(x\right)\\ =-d_{\max}^{2}\left(x\right)-x^{T}Mx-u^{*T}Nu^{*}+d^{T}\left(x\right)N\;d\left(x\right)-d^{T}\left(x\right)N\;d\left(x\right)-2u^{*}{\;}^{T}N\;d\left(x\right)\\ =-d_{\max}^{2}\left(x\right)+d^{T}\left(x\right)N\;d\left(x\right)-x^{T}Mx-{\left(u^{*}+d\left(x\right)\right)}^{T}N\left(u^{*}+d\left(x\right)\right)\\ \leq -d_{\max}^{2}\left(x\right)+d^{T}\left(x\right)N\;d\left(x\right)-x^{T}Mx. \end{array}$$

According to the condition given in Lemma 1, i.e., *d*_max_^2^(*x*) ≥ *d*^*T*^(*x*)*N*  *d*(*x*), we obtain

(9) $$\begin{array}{c} {\dot{V}}^{*}\left(x\right)\leq -x^{T}Mx, \end{array}$$

i.e., ${\dot{V}}^{*}\left(x\right)<0,x\neq 0$, and ${\dot{V}}^{*}\left(0\right)=0$, and the uncertain system (1) is asymptotically stable for any uncertainties *b*(*x*) and *d*(*x*). According to the above facts, the optimal solution *u*^*∗*^ is the robust control solution of the uncertain system (1). This completes the proof.

From Lemma 1, we have that if we select *N*=*I*_*m*_, where *I*_*m*_ is the identity matrix with *m* × *m*, then *d*_max_^2^(*x*) ≥ *d*^*T*^(*x*)*N*  *d*(*x*)=*d*^*T*^(*x*)*d*(*x*)=‖*d*(*x*)‖^2^ holds.

**Remark 1 .**

Lemma 1 shows that the robust control problem of the original uncertain system can be equivalent to the optimal control problem of the nominal system, and then, the solution of the robust control problem can be obtained indirectly by solving the optimal control problem. Therefore, this equivalence relationship can be used to develop a new robust control design method and solve it by using ADP method, as described in the following section.

**Remark 2 .**

It is well-known that *H*_*∞*_ control belongs to robust control. Although many *H*_*∞*_ control design techniques have been proposed, it should be noted that, as explained in Section 8.5 in [18], the *H*_*∞*_ control differs in the optimal method proposed in this paper. In the optimal control method, we start from the uncertainty bounds and then design the controller according to these bounds. Hence, if the controller exists, we can say the uncertain system is robustly stable.

## 3. Solving the Robust Control Problem via Adaptive Critic Learning

**Figure 1:**
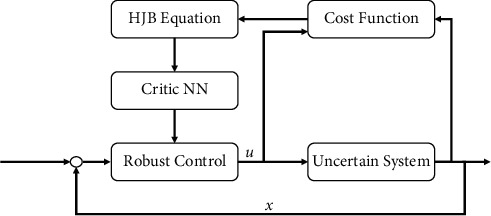
Schematic of the proposed control system.

To obtain the optimal control solution (7), the unknown cost function (5) should be resolved. However, it is quite difficult to address the cost function (5) directly; then, a critic NN in this section will be proposed to approximate the cost function (5); this allows to develop an adaptive learning method to update online the NN weights, where the convergence of NN weights can be retained. Because its strong convergence, the actor NN widely used in the ADP schemes is removed. The proposed control system structure is given in Figure 1.

This section will propose an adaptive critic learning method to obtain the solution of the derived optimal control problem. To this end, a critic NN is trained to estimate the cost function *V*^*∗*^(*x*), where the cost function *V*^*∗*^(*x*) is considered as continuous; hence, we have the following NN [13],

(10) $$\begin{array}{c} V^{*}\left(x\right)=W^{T}\sigma \left(x\right)+\varepsilon_{v}\left(x\right), \end{array}$$

where *W* ∈ *ℝ*^*l*^ is the ideal critic NN weight, *σ*(*x*) ∈ *ℝ*^*l*^ is the regressor vector, *l* is the number of neurons, and *ε*_*v*_(*x*) is the approximate error of NN.

Then, we have the partial derivative of cost function as

(11) $$\begin{array}{c} V_{x}^{*}\left(x\right)={\left(\nabla \sigma \left(x\right)\right)}^{T}W+\nabla \varepsilon_{v}\left(x\right), \end{array}$$

where ∇*σ*(*x*)=∂*σ*(*x*)/∂*x* ∈ *ℝ*^*l*×*n*^ is the regressor matrix and ∇*ε*_*v*_(*x*)=∂*ε*_*v*_(*x*)/∂*x* ∈ *ℝ*^*n*^ is the NN error.

Without loss of generality, the following assumption is given in [13].

**Assumption 2 .**

The NN weight *W*, the regressor vector *σ*, the regressor matrix ∇*σ*(*x*), and the approximate errors *ε*_*v*_(*x*) and ∇*ε*_*v*_(*x*) are all bounded, i.e., ‖*W*‖ ≤ *W*_*M*_, ‖*σ*‖ ≤ *σ*_*N*_, ‖∇*σ*‖ ≤ *σ*_*M*_, and ‖∇*ε*_*v*_‖ ≤ *σ*_*ε*_ for positive constants *W*_*M*_, *σ*_*N*_, *σ*_*M*_, and *σ*_*ε*_.

In fact, the ideal NN weight *W* is unknown; hence, we have that the practice $\hat{W}$ can be online updated; then, the actual cost function can be written as

(12) $$\begin{array}{c} \hat{V}\left(x\right)={\hat{W}}^{T}\sigma \left(x\right). \end{array}$$

Hence, the practical estimated optimal solution can be solved as

(13) $$\begin{array}{c} {\hat{V}}_{x}\left(x\right)={\left(\nabla \sigma \left(x\right)\right)}^{T}\hat{W}. \end{array}$$

According to (10) and (11), we have the ideal optimal control (7) as

(14) $$\begin{array}{c} u^{*}=-\frac{1}{2}N^{-1}g^{T}\left[{\left(\nabla \sigma \left(x\right)\right)}^{T}W+\nabla \varepsilon_{v}\left(x\right)\right], \end{array}$$

and its practical optimal control is given as

(15) $$\begin{array}{c} \hat{u}=-\frac{1}{2}N^{-1}g^{T}{\left(\nabla \sigma \left(x\right)\right)}^{T}\hat{W}. \end{array}$$

The problem next to be solved is solving the unknown weight $\hat{W}$, which can guarantee the stability of the controlled system and realize the convergence to the ideal value *W*. Most existing ADP methods can only get the uniform ultimate boundedness (UUB) of the approximated NN weight rather than the convergence. In this paper, a novel adaptive critic learning method is introduced to guarantee the convergence of $\hat{W}$ to *W*. This strong convergence property is conducive to avoiding the use of actor NN, and the optimal control approximated via critic NN can converge to its ideal optimal solution.

Substituting (11) into (4), we can rewrite the HJB equation as

(16) $$\begin{array}{c} 0=W^{T}\nabla \sigma \left(x\right)\left(f\left(x\right)+g\left(x\right)u\right)+d_{\max}^{2}\left(x\right)+x^{T}Mx+u^{T}Nu+\varepsilon_{\text{HJB}}, \end{array}$$

where *ε*_HJB_=(∇*ε*_*v*_(*x*))^*T*^[*f*(*x*)+*g*(*x*)*u*] is the residual error determined by the approximation error ∇*ε*_*v*_(*x*).

For developing an adaptive critic learning law to estimate the critic NN weight *W*, the known terms in (16) can be defined as

(17) $$\begin{array}{c} \left\{\begin{array}{c} \Xi =\nabla \sigma \left(x\right)\left[f\left(x\right)+g\left(x\right)u\right],\\ \Theta =d_{\max}^{2}\left(x\right)+x^{T}Mx+u^{T}Nu. \end{array}\right) \end{array}$$

Then, the HJB equation (16) with (17) can be given as

(18) $$\begin{array}{c} \Theta =-W^{T}\Xi -\varepsilon_{\text{HJB}}. \end{array}$$

According to (18), only the NN weight *W* is unknown in this parameterized formulation. Hence, it can be estimated by using a recently proposed learning algorithm [19, 20], which is driven by the derived estimation error.

To this end, the filtered regressor matrices *P* ∈ *ℝ*^*l*×*l*^ and *Q* ∈ *ℝ*^*l*^ can be denoted as [19, 20]

(19) $$\begin{array}{c} \left\{\begin{array}{c} \dot{P}=-\ell P+\Xi \Xi^{T},\;P\left(0\right)=0,\\ \dot{Q}=-\ell Q+\Xi \Theta ,\;\Theta \left(0\right)=0, \end{array}\right) \end{array}$$

where *ℓ* > 0 is a positive parameter. Hence, its solution can be derived as

(20) $$\begin{array}{c} \left\{\begin{array}{c} P=\int_{0}^{t}e^{-\ell \left(t-s\right)}\Xi \left(s\right)\Xi^{T}\left(s\right)\text{d}s,\\ Q=\int_{0}^{t}e^{-\ell \left(t-s\right)}\Xi \left(s\right)\Theta \left(s\right)\text{d}s, \end{array}\right) \end{array}$$

which can be online calculated based on the system state *x*.

With *P* and *Q* in (20), an auxiliary vector *M*_1_ ∈ *ℝ*^*l*^ can be defined as

(21) $$\begin{array}{c} M_{1}=-P\hat{W}+Q. \end{array}$$

From (18) and (20), we have *Q*=−PW+*v* with *v*=−∫_0_^*t*^*e*^−*ℓ*(*t* − *s*)^*ε*_HJB_(*s*)Ξ(*s*)d*s*. A bounded variable, e.g., ‖*v*‖ ≤ *ε*_*v*_, for a positive constant, *ε*_*v*_ > 0. Then, we can obtain from (19)–(21) that

(22) $$\begin{array}{c} M_{1}=-P\tilde{W}+v, \end{array}$$

with $\tilde{W}=W-\hat{W}$ being the NN weight estimation error.

The estimation error used in the adaptive learning algorithm can help to guarantee the convergence of the estimate, as shown in [13]. Hence, we can design the following adaptive law to online calculate $\hat{W}$ as

(23) $$\begin{array}{c} \dot{\hat{W}}=-\Gamma M_{1}, \end{array}$$

with Γ > 0 being the adaptive learning gain.

**Remark 3 .**

The adaptive law (23) is driven by the estimation error $\tilde{W}$. The purpose of this new learning algorithm is to guarantee the convergence of estimate $\hat{W}$ to unknown weight *W*. Therefore, the learning algorithm given in this paper is different from those used in the existing ADP methods, e.g., [3, 21], which employ the gradient-based methods [22] to guarantee the boundedness of $\hat{W}$ only.

To illustrate the convergence of the proposed learning algorithm, the positive definiteness of the matrix *P* defined in (20) will be introduced:

**Lemma 2 .**

When the regressor Ξ in (18) fulfills the persistent excitation (PE) condition, the matrix *P* defined in (20) is positive.

The convergence of the proposed learning algorithm can be summarized as follows.

**Theorem 1 .**

For the adopted critic NN with adaptive law (23), if the regressor vector Ξ in (18) satisfies the PE condition, the critic NN weight error $\tilde{W}$ exponentially converges to a small bounded set around zero.

**Proof**

For Lemma 2, the matrix *P* is positive definite when the regressor Ξ satisfies the PE condition, i.e., the minimum eigenvalue *λ*_min_(*P*) > *δ* > 0. Hence, a Lyapunov function can be chosen as $V_{1}=1/2{\tilde{W}}^{T}\Gamma^{-1}\tilde{W}$, and its derivative ${\dot{V}}_{1}$ along with (23) can be derived as

(24) $$\begin{array}{c} {\dot{V}}_{1}={\tilde{W}}^{T}\Gamma^{-1}\dot{\tilde{W}}=-{\tilde{W}}^{T}P\tilde{W}+{\tilde{W}}^{T}v, \end{array}$$

which further implies

(25) $$\begin{array}{c} {\dot{V}}_{1}={\tilde{W}}^{T}\Gamma^{-1}\tilde{W}+{\tilde{W}}^{T}v\leq -\left\|\tilde{W}\right\|\left(\delta \left\|\tilde{W}\right\|-\varepsilon_{v}\right). \end{array}$$

Thus, we have that the estimation errors of NN weight $\tilde{W}$ will converge to a compact set Ω: $\left\{\tilde{W}|\|\tilde{W}\leq \varepsilon_{v}/\delta \right\}$, in which the size of this set depends on the approximation error *ε*_*v*_ and the excitation level *δ*, i.e., for an arbitrarily small NN approximation error (according to the NN approximation property, this error can be arbitrarily small for sufficient NN nodes, i.e., ∇*ε*_*v*_(*x*)⟶0 with *l*⟶*∞*). Therefore, $\hat{W}$ can converge to *W*. In the ideal case, i.e., *ε*_HJB_=0 and *v*=0, then we know the estimation errors of weights $\tilde{W}$ converge to zero exponentially.

For system (2) with practical optimal control (15) and adaptive law (23), if the regressor Ξ satisfies the PE condition, the error $\tilde{W}$ converges to a small set around zero. Moreover, the actual optimal control *u* in (15) converges to a region around its optimal solution *u*^*∗*^ in (14), i.e., ‖*u* − *u*^*∗*^‖ ≤ *ε*_*u*_. Hence, the original robust control problem is resolved.

## 4. Simulation

### 4.1. Numerical Simulation

Consider an uncertain system as

(26) $$\begin{array}{c} \dot{x}=f\left(x\right)+g\left(x\right)\left(u+b\left(x\right)u\right)+g\left(x\right)d\left(x\right), \end{array}$$

where $f\left(x\right)=\left[\begin{array}{c} -x_{1}+x_{2}\\ -0.5x_{1}-0.5x_{2}\left(1-{\left(\cos\left(2x_{2}\right)+2\right)}^{2}\right) \end{array}\right]$, $g\left(x\right)=\left[\begin{array}{c} 0\\ \cos\left(2x_{1}\right)+2 \end{array}\right]$, $x={\left[\begin{array}{cc} x_{1}, & x_{2} \end{array}\right]}^{T}\in {\mathbb{R}}^{2}$ is the system state, *u* ∈ *ℝ* is the control input, and the term *k*(*x*)=*r*_2_*x*_2_^2^ with *r*_2_ ∈ [0,1] and *d*(*x*)=*r*_1_*x*_1_sin(*x*_2_) with *r*_1_ ∈ [−1,1] denote the uncertainties.

Because the uncertain terms *b*(*x*)=*r*_2_*x*_2_^2^ ≥ 0 and *d*(*x*)=*r*_1_*x*_1_sin(2*x*_2_) are bounded by ‖*d*(*x*)‖=|*p*_1_*x*_1_sin(2*x*_2_)| ≤ |*x*_1_|=*d*_max_(*x*), then we can obtain the optimal control problem as

(27) $$\begin{array}{c} \dot{x}=f\left(x\right)+g\left(x\right)u, \end{array}$$

with the cost function as

(28) $$\begin{array}{c} V\left(x\right)=\int_{t}^{\infty }\left(d_{\max}^{2}\left(x\right)+x^{T}Mx+u^{T}Nu\right)\text{d}s. \end{array}$$

As given in [18], the optimal cost function is written as

(29) $$\begin{array}{c} V^{*}\left(x\right)=x_{1}^{2}+2x_{2}^{2}. \end{array}$$

Then, we can obtain its optimal solution as

(30) $$\begin{array}{c} u^{*}=-\frac{1}{2}N^{-1}g^{T}V_{x}^{*}=-\left(\cos\left(2x_{1}\right)+2\right)2x_{2}. \end{array}$$

A critic NN will be used to approximate the cost function *V*; thus, the activation function *σ*(*x*) is defined as

(31) $$\begin{array}{c} \sigma \left(x\right)={\left[x_{1}^{2},x_{1}x_{2},x_{2}^{2}\right]}^{T}. \end{array}$$

To realize the simulation, we set the learning parameters Γ=100 and *ℓ*=10, the initial system state is given as $x_{0}={\left[\begin{array}{cc} 1, & -0.2 \end{array}\right]}^{T}$, the initial weight $\hat{W}\left(0\right)=0$, and the weight matrices are set as *M*=*I* and *N*=*I*.

**Figure 2:**
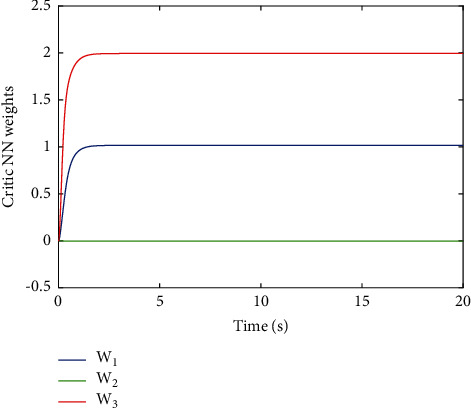
Convergence of the critic NN weights $\hat{W}$ [13].

**Figure 3:**
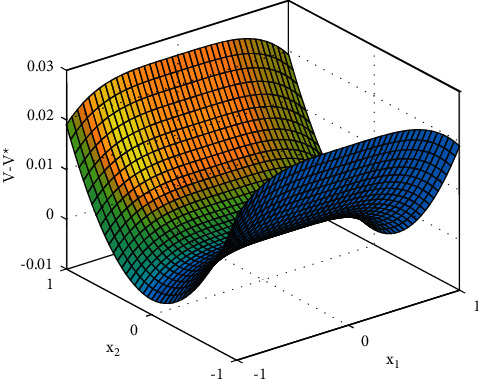
Critic NN approximation error of optimal cost function [13].

**Figure 4:**
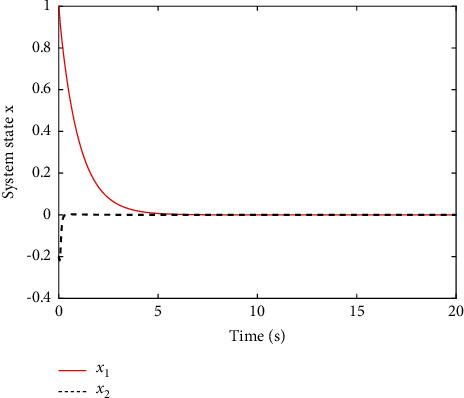
Profile of system state *x*.

**Figure 5:**
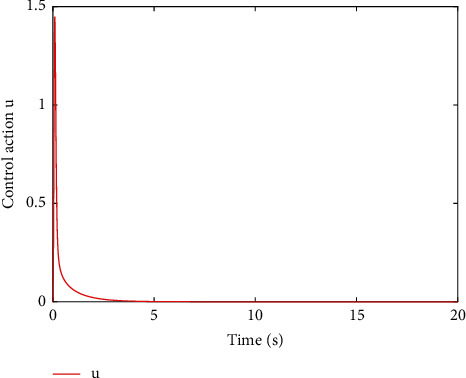
The system input for proposed control.

Figure 2 shows the estimated value of the evaluation NN weights. It can be seen from Figure 2 that the estimated NN weight converges to a certain value. This result verifies the convergence of Theorem 1 and the effectiveness of the proposed learning algorithm, which indicates that estimated critic NN weight $\hat{W}$ converges to its true value, i.e., $W=\left[\begin{array}{ccc} 1, & 0, & 2 \end{array}\right]$. To better display the performance of the proposed learning method, the error between the ideal cost function *V*^*∗*^ and practical coat function $\hat{V}$ is given in Figure 3, where we can obtain the fairly satisfactory approximation performance. In fact, the simulation results in Figures 2 and 3 can be also found in [13]; different from [13], this paper considers the uncertainties involved in control input.  Figure 4 shows the change of the state of the controlled system under the derived optimal control, which shows that the closed-loop system is asymptotically stable. The corresponding control input is shown in Figure 5, bounded and smooth.

### 4.2. Application to Robotic Systems

This section will develop a simulation based on a 2-DOF robot [18, 23]. To realize the simulation, the robotic systems model can be defined as

(32) $$\begin{array}{c} \bar{M}\left(q\right)\ddot{q}+C\left(q,\dot{q}\right)+F\left(\dot{q}\right)+G\left(q\right)=\tau , \end{array}$$

where *q* is the joint variables, *τ* is the generalized forces, $\bar{M}\left(q\right)$ denotes the inertia matrix, $C\left(q,\dot{q}\right)$ is the centripetal vector, *F*(*q*) is the friction vector, and *G*(*q*) defines the gravity vector. In this section, we denote $\bar{W}\left(q,\dot{q}\right)=C\left(q,\dot{q}\right)+F\left(\dot{q}\right)+G\left(q\right)$. There are uncertainties in $\bar{M}\left(q\right)$ and $\bar{W}\left(q,\dot{q}\right)$ due to the unknown load on the manipulator and unmodeled frictions.

The inertia matrix can be derived as

(33) $$\begin{array}{c} \bar{M}\left(q\right)=\left[\begin{array}{cc} {\bar{M}}_{11} & {\bar{M}}_{12}\\ {\bar{M}}_{21} & {\bar{M}}_{22} \end{array}\right], \end{array}$$

where ${\bar{M}}_{11}=J_{1}+J_{2}+m_{1}r_{1}^{2}+m_{2}l_{1}^{2}+m_{2}r_{2}^{2}+2m_{2}l_{1}r_{2}\cos\left(q_{2}\right)+m_{L}l_{1}^{2}+m_{L}l_{2}^{2}+2m_{L}l_{1}l_{2}\cos\left(q_{2}\right)$, ${\bar{M}}_{12}=J_{2}+m_{2}r_{2}^{2}+m_{2}l_{1}r_{2}\cos\left(q_{2}\right)+m_{L}l_{2}^{2}+m_{L}l_{1}l_{2}\cos\left(q_{2}\right)$, and ${\bar{M}}_{22}=J_{2}+m_{2}r_{2}^{2}+m_{L}l_{2}^{2}$.

The centripetal vector is

(34) $$\begin{array}{c} C\left(q,\dot{q}\right)=\left[\begin{array}{c} C_{1}\\ C_{2} \end{array}\right], \end{array}$$

where $C_{1}=\left(m_{2}l_{1}r_{2}+m_{L}l_{1}l_{2}\right)\left(2{\dot{q}}_{1}-{\dot{q}}_{2}\right){\dot{q}}_{2}\sin\left(q_{2}\right)$ and $C_{2}=\left(m_{2}l_{1}r_{2}+m_{L}l_{1}l_{2}\right){\dot{q}}_{1}^{2}\sin\left(q_{2}\right)$.

The friction vector and gravity matrix are

(35) $$\begin{array}{c} F\left(\dot{q}\right)=\left[\begin{array}{c} b_{1}{\dot{q}}_{1}\\ b_{2}{\dot{q}}_{2} \end{array}\right],G\left(q\right)=\left[\begin{array}{c} G_{1}\\ G_{2} \end{array}\right], \end{array}$$

where *G*_1_=(*m*_1_*gr*_1_+*m*_2_*gr*_1_+*m*_*L*_*gr*_1_)sin(*q*_1_)+(*m*_2_*gr*_2_+*m*_*L*_*gl*_2_)sin(*q*_1_+*q*_2_) and *G*_2_=(*m*_2_*gr*_2_+*m*_*L*_*gl*_2_)sin(*q*_1_+*q*_2_).

Some model parameters are given as *m*_1_=9.387/kg, *m*_2_=1.729/kg, *l*_1_=*l*_2_=0.250/m, *b*_1_=*b*_2_=0.14, *r*_1_=0.053/m, *r*_2_=0.170/m, *J*_1_=0.1190/kg.m^2^, *J*_2_=0.0652/kg.m^2^, *m*_*L*_=[0,3]kg, and *g*=9.8m/s. With above system dynamics, the state equation of the system can be given as [18]

(36) $$\begin{array}{c} \dot{x}=f\left(x\right)+g\left(x\right)\left(u+b\left(x\right)u\right)+g\left(x\right)d\left(x\right), \end{array}$$

where *x*=[*x*_1_, *x*_2_, *x*_3_, *x*_4_]^*T*^=[*q*_1_, *q*_2_, *q*_3_, *q*_4_]^*T*^, $f\left(x\right)=\left[\begin{array}{c} 0\\ x_{2} \end{array}\right],g\left(x\right)=\left[\begin{array}{c} 0\\ 1 \end{array}\right]$, $b\left(x\right)=\bar{M}{\left(x_{1}\right)}^{-1}{\bar{M}}_{0}\left(x_{1}\right)-I\geq 0$, ${\bar{M}}_{0}$ is the value of $\bar{M}$ when the load is 0. $d\left(x\right)=\bar{M}{\left(q\right)}^{-1}\left({\bar{W}}_{0}\left(q,\dot{q}\right)-\bar{W}\left(q,\dot{q}\right)\right)$, and ${\bar{W}}_{0}$ is the value of $\bar{W}$ when the load 0.

In this simulation, we set the initial weight $\hat{W}\left(0\right)=0$; when the load *m*_*L*_=3 kg, we choose the leaning parameters *ℓ*=21 and Γ=0.1 and weight matrices *M*=*I* and *N*=*I*. The initial states are *q*_1_(0)=15°, *q*_2_(0)=15°, ${\dot{q}}_{1}\left(0\right)=0$, and ${\dot{q}}_{2}\left(0\right)=0$.

**Figure 6:**
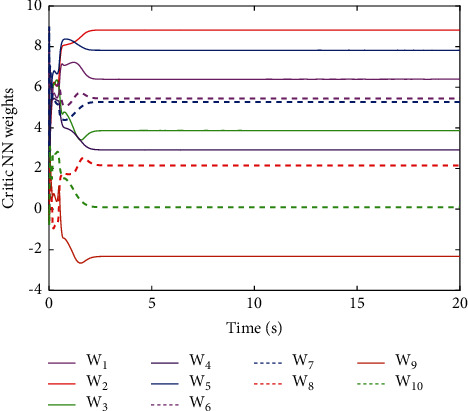
Convergence of the critic NN weights $\hat{W}$.

**Figure 7:**
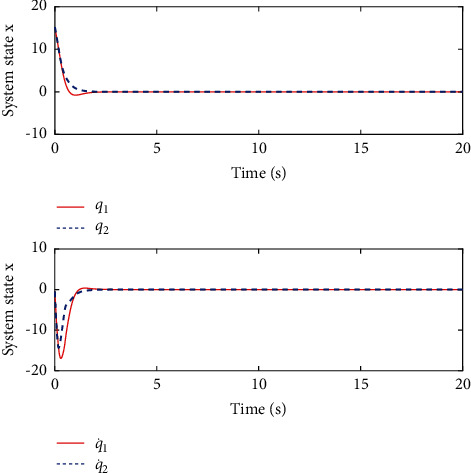
Profile of system state *x*.

**Figure 8:**
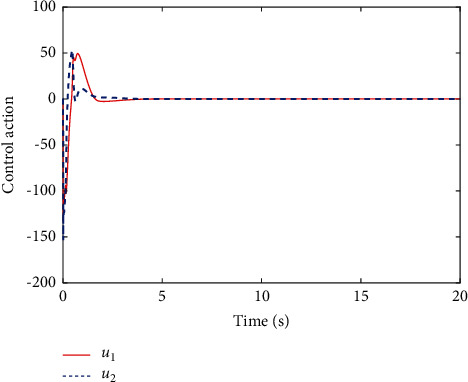
The system input for proposed control.

Figure 6 shows the estimated critic NN weights. It can be seen from Figure 6 that the estimated NN weight converges to certain value. This result verifies the convergence of Theorem 1 and the effectiveness of the proposed learning algorithm.  Figure 7 shows the change of the controlled system state under the derived optimal control when the load condition is set *m*_*L*_=3 kg, which shows that the closed-loop system is asymptotically stable. The corresponding control input is shown in Figure 8. Although it jitters at first, it tends to be smooth when it stabilizes.

From above simulation results, we have that the proposed learning method and control technique are effective.

## 5. Conclusion

The purpose of this paper is to address the robust control problem of the uncertain systems via developing an adaptive critic learning method. To this end, the robust control problem of the uncertain systems is transformed into an optimal control problem of the nominal systems via selecting the cost function. Then, a single NN is used to reformulate the cost function, where the unknown cost function can be represented as a known term; then, an adaptive critic learning method based on the adaptive parameter estimation technique is presented to obtain the optimal cost function such that the optimal control problem can be solved. Simulations are given to show the effectiveness of the proposed leaning algorithm and control method. Future work will focus on the robust tracking control with uncertain systems.

## Data Availability

Data were curated by the authors and are available upon request.
